# Students as carbon accountants: calculating carbon costs of a PhD in neuroscience

**DOI:** 10.1093/genetics/iyaf268

**Published:** 2025-12-17

**Authors:** William V Smith, Aimee Bebbington, Ranjini Sircar, Malte C Gather, Stefan R Pulver

**Affiliations:** School of Psychology and Neuroscience, University of St Andrews, St Mary's Quad, South Street, St Andrews KY16 9JP, United Kingdom; School of Mathematics and Statistics, University of St Andrews, St Andrews KY16 9SS, United Kingdom; Biomedical Sciences Research Complex, University of St Andrews, St Andrews KY16 9ST, United Kingdom; Centre of Biophotonics, University of St Andrews, St Andrews KY16 9SS, United Kingdom; School of Psychology and Neuroscience, University of St Andrews, St Mary's Quad, South Street, St Andrews KY16 9JP, United Kingdom; Humboldt Centre for Nano- and Biophotonics, Department of Chemistry, University of Cologne, Greinstr. 4-6, Cologne 50939, Germany; Centre of Biophotonics, University of St Andrews, St Andrews KY16 9SS, United Kingdom; Humboldt Centre for Nano- and Biophotonics, Department of Chemistry, University of Cologne, Greinstr. 4-6, Cologne 50939, Germany; SUPA, School of Physics and Astronomy, University of St Andrews, St Andrews KY16 9SS, United Kingdom; Institute for Behavioural and Neural Sciences, University of St Andrews, St Mary's Quad, South Street, St Andrews KY16 9JP, United Kingdom; School of Psychology and Neuroscience, University of St Andrews, St Mary's Quad, South Street, St Andrews KY16 9JP, United Kingdom; Centre of Biophotonics, University of St Andrews, St Andrews KY16 9SS, United Kingdom; Institute for Behavioural and Neural Sciences, University of St Andrews, St Mary's Quad, South Street, St Andrews KY16 9JP, United Kingdom

**Keywords:** sustainability, neurogenetics, carbon footprint, flies, insects

## Abstract

Research is an energy- and resource-demanding activity. However, despite emerging sustainability initiatives, a paucity of data and uptake of green initiatives continue to hamper effective and accountable emissions mitigation. Worldwide, >250,000 doctoral students graduate annually across all academic disciplines. Empowering students to engage in carbon accounting can raise awareness of sustainability in research and provide a substantial and robust resource of carbon data alongside a powerful community-driven impetus for decarbonization. Here, we demonstrate how students and other researchers can consistently measure the carbon footprint of their work, using 1 PhD student's research in a *Drosophila* neuroscience lab as our case study. We present a life cycle assessment of the equivalent carbon dioxide emissions generated by the student's research activities. Moreover, we explain how students can create a “carbon appendix’ to their research, as a common framework for disseminating carbon data and revealed strategies for improving research sustainability. We argue that the process of creating a carbon appendix can empower researchers to scrutinize sustainability practices, empower them to implement effective green initiatives, and identify data-driven solutions to meet and exceed funders' sustainability targets.

## Teaser

Research is energy intensive. Here Smith et al. describe methods for ascertaining the carbon footprint of PhD research in a neuroscience laboratory and propose a framework for creating Carbon Appendices which can be used to document carbon emissions across research disciplines.

## Introduction

Greenhouse gas emissions resulting from human activities are a major driver of global warming and climate change. Efforts to systematically reduce and mitigate the effects of excess atmospheric greenhouse gases involve coordinated actions across all sectors of life. Scientific research is 1 domain of the carbon economy that may come to increasingly dominate the landscape of energy usage in the coming decades. As the frontier of scientific knowledge expands, the complexity of the problems we address rises. The advancement in high-energy experimental techniques, the exponential rise of, and dependency on, artificial intelligence (AI) in analytical pipelines, and the increasing number of scientific laboratories across the world mean the carbon burden of science will continue to rise as we approach our climate targets (Budennyy et al. 2022; Tamburrini 2022). Within this context, scientific researchers are increasingly considering the environmental impacts of their work. Indeed, research funding bodies are placing expectations on researchers when funding research to recognize and engage in shared action to eliminate one's negative environmental impacts and achieve the transition to sustainable practices.

By focusing on “cradle-to-grave” life cycle assessments, institutions (McGain and Naylor 2014; Valls-Val and Bovea 2021; Martin et al. 2022) and individual researchers (Thiel et al. 2017; Gordon et al. 2021; Elli et al. 2024) have begun to account for and mitigate their scope 1 (direct release), scope 2 (indirect via off-site energy production), and scope 3 (all other sources up- and downstream of research activities) carbon emissions. At the individual level, researchers have contributed to estimating the carbon associated with specific resources (e.g. petri dishes and Pasteur pipettes in Farley and Nicolet 2023) and domain-specific practices such as genome-wide association studies (GWAS) for bioinformatics (Grealey et al. 2022) and statistical analysis of functional magenetic resonance imaging (fMRI) data (Souter et al. 2025). Individual-led action in computation (Lannelongue et al. 2021) and clinical sciences (Farley and Nicolet 2023) has demonstrated the power of researchers in generating carbon footprint data for a variety of specialized domains. Such actions have supported collective action by researchers to design laboratory sustainability education and accreditation programs, including the Laboratory and Efficiency Assessment Framework (LEAF) and My Green Lab (Schell and Bruns 2024). At the institutional level, organizations across the world have devised data-driven strategies to reduce the carbon footprint of sectors, including healthcare (Purohit et al. 2021; Robinson et al. 2023; Maida et al. 2024), agriculture (Mazzetto et al. 2022; Ozlu et al. 2022), and industrial manufacturing (Panagiotopoulou et al. 2022), while universities have spearheaded efforts to account for and mitigate the carbon footprint of education- and research-related travel (Arsenault et al. 2019; Yañez et al. 2020).

However, the conception and implementation of more sustainable practices remain challenging due to gaps in our understanding of carbon costs across diverse research methodologies, limited human capital, and the diversity of approaches to calculating and reporting emissions, which can make individuals and institutions reluctant to engage in carbon accounting (Fenner et al. 2018; Robinson et al. 2018). While these challenges do call for a broad synthesis of current data and best practice, widespread implementation and expansion of current knowledge require awareness, engagement, and confidence within the next generation of changemakers.

We propose empowering early-career researchers, particularly PhD students, to measure and report the carbon footprint of their research as a powerful way to cultivate awareness of, engagement with, and confidence in navigating the research sustainability landscape. Specifically, we present the notion of “carbon appendices” to PhD theses as a consistent reporting venue, in which students can present their methodology, emissions estimates, and recommendations for improving the sustainability of their work. With 115,705 PhD students studying in the United Kingdom alone in 2022 to 2024 (HESA 2023), and an estimated 277,700 doctorates awarded annually worldwide across all disciplines (OECD 2019), carbon appendices could provide a wealth of openly accessible, rigorously scrutinized carbon data and sustainability recommendations across a wide variety of research methods. Given that human capital restraints are 1 hurdle to advancing sustainability (Blanco-Portela et al. 2017; Álvarez Jaramillo et al. 2019; Leal Filho et al. 2019; Veiga Ávila et al. 2019), even small contributions at an individual student level could enable significant positive change for individual labs and institutional departments. Expanding the concept of a carbon appendix to published manuscripts could extend the reach of this impact. Moreover, as many graduate students will become part of the next generation of academics and public servants, engaging students with carbon accounting could help strengthen commitment to sustainability within academia and industry in the long term.

Here, we demonstrate how students can consistently and efficiently measure the carbon footprint of their work, using 1 doctoral student's research in a neuroscience *Drosophila* lab as our case study. We estimate the carbon costs associated with each stage of the research life cycle, from procurement to experiments, analysis, data storage, and material disposal. As part of this life cycle assessment, we estimate carbon footprints for many activities relevant to other neuroscience and *Drosophila* labs, including scope 1 emissions from anesthetization of flies; scope 2 emissions from food preparation, commonly used experimental equipment and analytical pipelines, and waste disposal; and scope 3 emissions associated with fly requisitions from stock centers. We use these estimates to provide suggestions for how researchers employing similar techniques can improve the sustainability of their work. Finally, we discuss how uptake of carbon appendices could improve research sustainability, both by informing lab- and institutional-level emissions reduction strategies and by empowering students to confidently engage with sustainability initiatives within and beyond academia.

## Materials and methods

### Power estimation

The power usage of experimental and laboratory equipment was determined using RS Pro Energy Meters (Stock No. 178-5370; Model No. PM01; Stock No. 123-3230). The power demands of equipment associated with experiments (e.g. calcium imaging) were recorded for 1 to 3 instances of mock experiments by using the power meters interlocked from wall mains supply. The power of persistent equipment (e.g. incubators and fridges) was recorded over a 24- to 72-h period in February to March 2023. All equipment measurements were performed by using the power meters plugged between each equipment item and the mains electricity plug. We estimated the power usage of our analysis using the hardware analysis and monitoring software HWiNFO during analysis of experimental-related data (e.g. traces and Python-based visualization) (https://www.techspot.com/downloads/5245-hwinfo64.html). Clamp meters (Stock No. 123-3230) were used to measure energy from air conditioning (AC) units. The high-powered equipment for food generation and autoclaves precluded direct measurement for safety reasons; thus, we resorted to maximum power calculations based on the equipment's industry-reported metrics. The power usage of our laboratory spaces was monitored through direct building power meters reported by university-maintained SystemsLink and subsequently applied to our research based on floor area (electricity: 71.48 kWh/m^2^; gas: 170.14 kWh/m^2^). The mean power consumption readings (in kWh) were recorded for each equipment item (Table 1) and then converted into equivalent carbon dioxide (CO_2_e) values (see “Power-to-CO_2_e conversion”).

**Table 1.** Energy costs and model types for persistent and experimental equipment.

Lab sphere	Type	Equipment	kWh	Model
Persistent		Incubator (19 °C)	0.070	Panasonic MIR-554-PE
		Incubator (25 °C)	0.010	
		Incubator 25 L (19 °C)	0.010	Vevor Reptile Incubator
		Incubator 25 L (25 °C)	0.070	
		Fridge (10 °C)	0.100	LEC (model no. L550WS, serial no. 7D 0144847)
		Fridge/freezer (10 °C/−13 °C)	0.040	Liebherr Comfort
		Vial freezer (−20 °C)	0.400	Bush 43282301
		CO₂ monitoring system	0.010	Flamefast CellarGuard
		Gas heating system	8,796	
		Electricity system	2,138	
		Large AC system	0.644	Mitsubishi Electric AC/HP (MUZ-SF3SVE, 6 03468T)
		Small AC system	0.552	Daikin AC/HP (RXS50L2V1B) System
Virgin collection/preparation		LED	0.004	Euronex LE. 5208 AC100V-240V 90-7843
Saline preparation		Water purification	0.145	Millipore Q Water System F3J15741N + Suez Purite Deionizer
Experiments	Calcium imaging	Camera and manipulator	0.045	Andor iXon3 Camera (100 ms) and MPC-200, TH4-200 Olympus, and ROE-200_1849 Shutter
		Power supply	0.014	CAIRN OptoLED Power Supply 3666
		Perfusion system	0.002	Kamoer KCP3-BIOW
		Computer and interface	0.082	i7-8700 3.2 GHz CPU, 16 GB RAM, NVIDIA GeForce GTX 1080, and Interface NI-USB-6229 National Instruments Digital I/O System, AD PowerLab Instrument 16/35-PL3616-0154
	Electrophysiology and optogenetics	Camera and manipulator	0.045	Andor iXon3 Camera and MPC-200, TH4-200 Olympus Microscope Accessory + ROE-200_1849 Shutter Instrument Manipulator
		Power supply	0.014	CAIRN OptoLED Power Supply 3666
		Computer and interface	0.360	i7-8700 3.2 GHz CPU, 16 GB RAM, NVIDIA GeForce GTX 1080, and NI-USB-6229 National Instruments Digital I/O System, AD PowerLab Instrument 16/35-PL3616-0154
		Amplifier	0.007	AM System Differential AC Amplifier Model (#59721, 1700)
	Dual-color imaging	Camera	0.027	ORCA-Fusion C14440 Hamamatsu
		Spinning disk	0.006	Emission White Filter Spinning Disc CREST
		Light source	0.150	Lumencore
		Computer and interface	0.234	HP Z9, 2x Intel Xeon Silver 4215 CPU @ 2.5 GHz, 2,494 MHz, 8 cores, 16 logical processors, 192 GB RAM + Intel Screen
	Behavioral tracking	Computer and camera	0.054	HP i7-8700 3.2 GHz, 3,192 MHz, 6 cores, 12 logical processors, 16 GB RAM, and Navitar Zoom 7000
		Light stimulation	0.006	Thorlabs DC2200 + SOLIS-3L-light 600 mA constant stimulation for 10, 20, and 30 s
		Infra-red (IR) light	0.006	Andoer IR49S Mini IR
Analysis		Computer and interface	0.121	12th Intel i9-12900 HK, 14 cores, 20 logical processors, 64 GB RAM, NVIDIA GeForce RTX 3070 Ti GPU + Acer Monitor
Food generation		Cooking and mixer	7.536 (max)	Cleveland 13L
Vial disposal		Autoclave	10 (max)	Astell Scientific, SPBN21642, ASB270BT65

### Direct CO_2_ release from virgin collection

The collection of *Drosophila* virgins for genetic crosses necessitates anesthesia by direct application of CO_2_. *Drosophila* adults are initially anesthetized within their vial using a gas gun, then tipped onto a CO_2_ pad where CO_2_ is intermittently released to arrest movement. We estimated the direct release of CO_2_ during virgin collection from a gas gun (Scientific Laboratory Supplies [SLS] Flystuff Blowgun FLY1042) through a graduated 100-mL glass gas syringe (9 L/min) and from a CO_2_ pad (SLS Flystuff Flypad Standard FLY1208) using a fluid displacement method. Specifically, we submerged the gas pad in water, below an inverted funnel and measuring cylinder, and measured the time taken for gas released from the pad to displace the water inside the measuring cylinder. We timed the application of CO_2_ during virgin collection among 3 researchers to arrive at the average CO_2_ released per vial during virgin collection. We scaled up the measurement for heavy genetic screening laboratories based on reported vial load for 2 and 3 stage genetic constructs.

### Power-to-CO_2_e conversion

We utilized the open-source, freely available National Grid Energy System Operator (ESO)'s regional Carbon Intensity API to convert the power usage of equipment associated with experiments and persistent equipment to a valid CO_2_e estimation. For the 2023 to 2024 period, counts per month of experiments were recorded and translated into CO_2_e estimations using the API based on the custom-created Python code accessible in our data repository (see “Data availability”). Specifically, we took the mean of the first 2 weeks of each month (June 2023 to 2024) to calculate our monthly associated research CO_2_e emissions. The detailed methodology of the regional Carbon Intensity API is available on GitHub (https://carbon-intensity.github.io/api-definitions/#carbon-intensity-api-v2-0-0). Specifically, we assumed the mean power of all equipment to create a standard kWh for each experimental procedure. Based on monthly saved data, we tabulated the monthly total for each experimental type conducted and calculated the CO_2_e based on the API reporting. The code for data extraction from the API, processing, and visualization is available in our repository.

### Travel cost and procurement estimation

Estimates of CO_2_e travel costs were made using a combination of the UK government (UK GOV) 2024 reported conversion factors (https://www.gov.uk/government/publications/greenhouse-gas-reporting-conversion-factors-2024), the self-reporting by ScotRail (https://www.scotrail.co.uk/carbon-calculator), and the International Civil Aviation Organization (ICAO) Carbon Emissions Calculator (ICEC) (https://www.icao.int/environmental-protection/Carbonoffset/Pages/default.aspx). The methodology for each report is detailed through provided links. We used Google's Distance Matrix API to calculate the distance of journeys.

We estimated the CO_2_e emissions associated with equipment transport during procurement based on the reported site of manufacture. Nonrecyclable vials (FLY1312, SLS) are manufactured and assembled in Tijuana, Mexico. We aimed to estimate the range of associated CO_2_e emissions for procurement from Tijuana to St Andrews based on Distance Matrix API calculations and reported destinations for travel. We assumed that vial exports delivered by air freight came directly from Tijuana International Airport to Edinburgh Airport. We assumed that cargo-based exports came from the main Mexican export port, Manzanillo Port. We assumed direct travel between the reported final destination of manufacture and the final procurement destination. *Drosophila* fly stocks were procured from Bloomington, Indiana. We assumed air freight from Indianapolis International Airport to Edinburgh Airport. We assumed that vehicle transport of both vials and flies used an average Heavy Goods Vehicle (HGV) per the UK GOV reported emissions values.

## Results

### The carbon cost of *Drosophila* neuroscience research

**Fig. 1.**
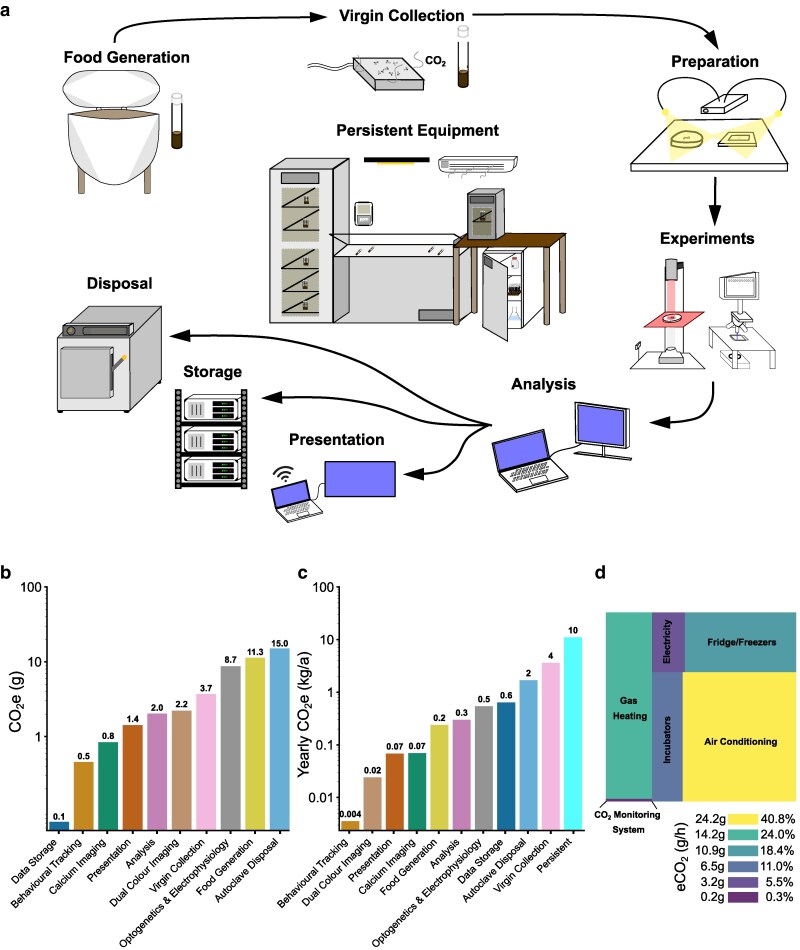
The carbon costs associated with 1 year of *Drosophila* neuroscience research. a) The life cycle of *Drosophila* experimental research. b) CO_2_e (g) emissions per individual experimental preparation (i.e. per larva) (data storage, behavioral tracking, calcium imaging, presentation, analysis, dual-color imaging, and optogenetics and electrophysiology), per vial (virgin collection), and per 700 medium-sized vials (food generation and disposal) to 1 d.p. CO_2_e estimates are based on 1 researcher's experimental and preparation time, with carbon costs calculated based on the kWh-to-CO_2_e conversion factor for the South Scotland National Grid (see “Materials and methods”). c) Annual CO_2_e (kg/a) emissions generated from the work of 1 student researcher in South Scotland for the period June 2023 to May 2024, including all aspects of the research life cycle based on the total number of experiments completed, vials used, and data generated per month. d) The average hourly CO_2_e (g/h) emission rate and percentage emissions contributions of different persistently working laboratory equipment, calculated from equipment energy usage and the average monthly kWh-to-CO_2_e conversion factor for South Scotland.

*Drosophila* neuroscience research generates scope 1, 2, and 3 carbon emissions. Over the course of 1 year, we conducted life cycle analyses that accounted for the carbon generated by all stages of 1 PhD student's (the first listed author's) research activities, assuming all energy came from the mains network grid in Southern Scotland. Here, we detail the measured and estimated CO_2_e emissions for each stage of the research life cycle in a chronological manner commencing with food generation and finishing with material disposal and data storage (Fig. 1a). We describe how we quantify carbon emissions from all stages of the research cycle. A summary of this quantification is provided in Fig. 1b and c. A checklist of relevant starting points for constructing similar life cycle assessments is provided in the supplementary information to the paper (see Supplementary Fig. 1).

#### Food generation

All animal research depends on food generation. This often relies on in-house equipment, which generates scope 2 carbon emissions via electricity consumption. For *Drosophila*, food sources consist of a ready-made cornmeal-based resource, which is rehydrated, heated, and mixed (Lakovaara 1969) Flies are raised on this medium in vials, with a new generation of flies transferred to fresh vials each month to maintain the genetic stock. In our laboratory, 216 stocks are maintained, and additional vials are used to rear flies for crosses and experiments. Every month, these activities for our research group consumed a combined 700 medium-sized vials with an average of 10 g of yeast extract food per vial. The individual PhD student in this study used on average 52 vials per month, amounting to 7.4% of the total laboratory consumption (see “Materials and methods”). In common with many laboratories, large batches of food are supplied across multiple research groups. Consequently, technicians rely on large-scale, high-powered equipment whose energy supply is not easily accessible. Modern research kitchen equipment often incorporates digital monitoring of energy consumption. However, in our case, the use of older equipment precluded direct determination of energy usage for safety reasons. Thus, we accounted for the maximum electrical power required for food generation based on manufacturer reporting. For heating and mixing of food, we found a maximum power usage of 7.536 kWh (Table 1). We estimated the associated greenhouse gas emissions from electricity generation using region- and time-specific kWh-to-CO_2_e conversion factors. The kWh-to-CO_2_e conversion factor varies across time and space due to changes in the balance of power derived from different types of power sources (Raugei et al. 2020) contributing dynamically to the UK National Grid. To find kWh-to-CO_2_e conversion factors for Southern Scotland, we used the Carbon Intensity API, a free resource that provides region-specific information on the carbon intensity of UK National Grid energy based on machine-learning forecasting (api.carbonintensity.org.uk). Using estimates of monthly average conversion factors (see “Materials and methods”), we found that food generation for the whole research group emits on average 152.4 g CO_2_e per month, which, given monthly variations, totaled 3.2 kg CO_2_e for the June 2023 to May 2024 period. Given that our individual PhD student utilized 7.4% of the total monthly vials, food generation for this single PhD student's work emits on average 11.3 g CO_2_e per month (Fig. 1b), which, given monthly variations, totaled 237.8 g CO_2_e (Fig. 1c) for the June 2023 to May 2024 period.

#### Drosophila genetics

*Drosophila* are commonly used model organisms in neuroscience due to ease of genetic selection involving selective breeding between transgenic lines. A vital part of this process is anesthetization of flies with CO_2_, resulting in scope 1 carbon emissions. Anesthetization involves the variable use of a CO_2_ gun (up to 2 s per vial) and pad (up to 10 s per vial). By directly collecting gas released from a CO_2_ gun and pad (see “Direct CO_2_ release from virgin collection”), we accounted for the direct release of 3.7 g CO_2_ per vial of sorted flies, where we specifically considered a simple collection of virgins for genetic crosses. It is worth noting that other collection procedures, for example those involving identification of multiple phenotypic markers, could conceivably release substantially more CO_2_. Considering the 2,074 active *Drosophila* laboratories as of December 2024 (https://wiki.flybase.org/wiki/FlyBase:Fly_Lab_List), the level of direct release of CO_2_ from anesthetization is likely large but currently unknown. For 1 PhD student, we calculated a total of 3.6 kg CO_2_e release (from 972 vials) from virgin collection per year (Table 2). However, *Drosophila* labs that rely more heavily on screening genetics (i.e. involving several, consistently used multistage genetic crosses like in developmental-based *Drosophila* research) could conservatively directly release <16.7 kg CO_2_e (∼4,500 vials) a year per researcher (see “Materials and methods”). Though this method of direct measurement can yield a reasonable estimate of CO_2_ released per vial of sorted flies, which can be scaled up to an annual estimate, labs may wish to consider the replacement rate of CO_2_ cylinders to obtain an estimate of direct CO_2_ release per researcher, where this information is available.

**Table 2.** CO_2_e research costs for 1 researcher in 2023 to 2024.

Task	Per task CO_2_e (g/h)	Yearly *N*	Time per task (h)	Yearly CO_2_e (g)
Virgin collection	1.60	972	∼	1,555.20
Preparation	0.18	132	∼	23.76
Behavioral tracking	0.40	8	2.5	3.53
Calcium imaging	0.80	94	1.0	69.34
Dual-color imaging	2.20	33	0.3	24.00
Optogenetics and electrophysiology	8.70	16	2.5	539.23

Lab task	CO_2_e (g/vial)	Yearly *N*	Time per task (h) (max vials)	Yearly CO_2_e (g)
Food generation	0.21	8,400	1 (400)	3,200.60
Vial disposal	1.47		2 (150)	2,240.00

The yearly CO_2_e (kg) is calculated on the average of the yearly South Scotland kWh/CO_2_e conversion with per task.

#### Preparation and experiments

Next, we wanted to understand how energy use during experimental work contributes to scope 2 carbon emissions. To estimate the carbon intensity of different experiments throughout the year, we used power meters to measure the power drawn by electrical apparatus during experiments (see Table 1 for the measured energy usage of different apparatus), retrospectively counted the amount of saved data for each experiment type, and applied monthly average kWh-to-CO_2_e conversion factors calculated from the Carbon Intensity API (api.carbonintensity.org.uk) data for the period June 2023 to May 2024 (see “Materials and methods”).

Experiments involving optogenetics, calcium imaging, and electrophysiology are staples of modern research especially in neuroscience (Knot et al. 2005; Scanziani and Häusser 2009; Häusser 2014). All of these experiments utilize high-powered light emitting diode (LED) lights, high-frame rates (100 frames per second), supercooled (−70 °C) cameras, and persistent signal processing units. In our work, we used these experimental techniques to monitor the motor activity in isolated *Drosophila* neural tissue using genetically encoded calcium indicators and motor root bursting patterns. In the case of electrophysiology and optogenetics, we used pulses of LED-generated light to induce changes in neural network activity, which we measured with continuously recording electrodes. For dual-color calcium imaging, we utilized a spinning disk confocal microscope to acquire images of neurons simultaneously expressing 2 different genetically encoded fluorescent calcium indicators.

All imaging and optogenetics experiments were performed on isolated neural tissue immersed in physiological saline and dissected under a dissecting microscope fitted with LEDs. Physiological saline (Baines External Saline; Marley and Baines 2011) required the use of deionization and filtration equipment that yielded 5.27 g CO_2_e for every 4 L of saline produced. Over the 2023 to 2024 research year, saline generation accounted for 26.37 g CO_2_e (20 L). Our microscope's LEDs yielded, on average, 0.03 g CO_2_e every preparation (up to 10 min per preparation) amounting to a precursory amount of 4.07 g CO_2_e for all experimental work (151 preparations). Overall, for dissection alone, we calculated an average annual carbon intensity of 30.44 g CO_2_e (Table 2). Preparation time was directly factored into each experimental estimation.

Based on the average kWh-to-CO_2_e conversion factor for 2023 to 2024, we found the average CO_2_e emissions per preparation for each technique (without preparation): behavioral optogenetics (0.5 g); calcium imaging (0.8 g); dual-color calcium imaging (2.2 g); and optogenetics and electrophysiology (8.7 g) (Fig. 1b). Over the course of the year June 2023 to May 2024, we recorded the quantity (151 experiments) and duration (154.5 h) of all experimental work performed by 1 PhD student (Table 2). Again, using monthly average kWh-to-CO_2_e conversion factors, we found that these experiments produced a total of 0.64 kg CO_2_e annually (Fig. 1c).

#### Data analysis, storage, and presentation

Next, we evaluated the emissions associated with data analysis, storage, and presentation.

Analysis requires computationally demanding and time-intensive processing pipelines as well as persistent data storage and maintenance that compound energy costs with data quantity and time. Within a year, the neuroscience PhD student utilized several different central processing unit (CPU)-based analytical pipelines to visualize and interpret recorded data. For calcium imaging, image sequences required positional stabilization over time, extraction and visualization of fluorescence changes in regions of interest, and relational analysis across many regions of interest. For electrophysiology, nerve root bursting signals require preprocessing rectification and smoothing followed by extracting signal relationships and quantities like bursting duration. For behavioral tracking, videos are analyzed using trained neural networks through the software TrackMate (Tinevez et al. 2017) and DeepLabCut (Mathis et al. 2018) to extract positional and velocity information over time. For the 2023 to 2024 research year, calcium imaging and electrophysiology were the only analytical pipelines utilized. All analyses required extraction and postprocessing with programs like DataView (Heitler 2007) and custom-built Python scripts. Through tracking the hourly power usage on a laptop during 5 instances of post hoc analysis (i.e. preprocessing in a signal processing software using DataView and postprocessing and visualization in Python 3), we accounted for an average of 2.0 g CO_2_e per preparation, yielding 302 g CO_2_e across the year.

All of the data collected were stored within a university-based secured remote server (PureSANs array) with power diagnostics reported weekly from the server provider. The combined power consumption of the server's production and the total flash disk storage was 34 MWh/year. We estimated the data storage energy cost based on the proportion of our laboratory's data share size (27.39 TB, 2.3% of the total server space [1,228.8 TB]) to be 19 kWh/TB/year, which equated to 512 kWh/year. Based on the South Scotland kWh-to-CO_2_e conversion factor (20 g CO_2_e per kWh; Fig. 1b), the emissions burden of the laboratory's data storage amounted to 1.2 g CO_2_e per hour or 10.35 kg CO_2_e for the 2023 to 2024 research year. A single year of PhD research generated 1.69 TB of data (i.e. raw video files, extracted csv files, Python scripts, figures, and manuscripts), equivalent to 6.2% of the laboratory's total data storage space. Thus, the emissions burden of data storage for a single South Scotland PhD student amounted to 0.07 g CO_2_e per hour (Fig. 1b) or 637.7 g CO_2_e for the 2023 to 2024 research year (Fig. 1c).

Beyond analysis and storage, data are often communicated, shared, and presented through video streaming applications (i.e. Microsoft Teams) to supervisors, academic staff, and wider academic circles. We found that 0.07 kWh was used by our laptop model (Table 1) per 1-h online meeting where visual and audio material was shared continuously amounting to, on average, 1.42 g CO_2_e per instance (Fig. 1b). Given our recorded average of 4 online contact hours per month, we accounted for 68 g CO_2_e for the 2023 to 2024 researched year (Fig. 1c).

Overall, data storage, analysis, and online presentations produced 1 kg CO_2_e for the 2023 to 2024 research year (1.6× the yearly CO_2_e generated from all recorded experimental work). Importantly, data storage would become a persistent and increasing carbon cost in future yearly accounts as the rise of open science demands retention of core datasets on publicly accessible, permanent servers.

#### Disposal

Frontier biological research using organisms often requires extensive use of energetically expensive sterilization and disposal mechanisms, thereby incurring a scope 2 carbon cost. At the end stage of the *Drosophila* research life cycle (Fig. 1a), high-temperature, high-pressure autoclaves are used to sterilize vials, glassware containing chemical modulatory compounds, and equipment that has contacted genetically modified material. We accounted for the 10-kWh maximum power usage specified by the manufacturer (Table 1), resulting in a maximum average of 202.2 g CO_2_e per month for the total laboratory requirement of 700 vials. We found that autoclaving produced a total of 22.7 kg CO_2_e for the June 2023 to May 2024 period for the entire laboratory. For a single PhD student (7.4% of the total load), autoclave disposal of vials amounted to 15.0 g CO_2_e per month (Fig. 1b) and a total of 1.7 kg CO_2_e (Fig. 1c) for the June 2023 to May 2024 period.

#### Persistent equipment

Accompanying the experimental life cycle, equipment for climate control (e.g. AC and incubators) is an essential and permanently on feature of research facilities. Within our 82.52 m^2^ research space, gas-based heating systems and national grid-supplied electricity for lights were used alongside AC units, incubators, CO_2_ monitoring systems, and fridge/freezer units (Table 1; Fig. 1d). The University of St Andrews uses SystemsLink to dynamically track kWh used for heating and lighting per floor area per building (see “Materials and methods”). Using SystemsLink information, we found building maintenance (e.g. heating and lighting) accounted for 36% of the total persistent equipment emissions. Surprisingly, incubators accounted for only 5.5% of the total persistent equipment CO_2_e emissions, producing on average ∼3.2 g CO_2_e an hour. The persistent CO_2_ monitoring systems yielded an average of 0.2 g CO_2_e an hour. Overall, persistent equipment accounted for the largest proportion of CO_2_e generated per year in our laboratories (212 kg CO_2_e; Fig. 1c).

Taken together, 1 year of *Drosophila* cellular neuroscience research conducted by a PhD student in South Scotland in 2023 to 2024 produced 18.15 kg CO_2_e. In particular, across the research life cycle, direct research-related activities generated 3.62 kg scope 1 (i.e. direct release by virgin collection and gas-related heating), 13.84 kg scope 2 (i.e. food generation, experimental work, analytical pipelines, and resource disposal), and 0.71 kg scope 3 (i.e. data storage, distribution, and presentation) CO_2_e emissions costs. Such reporting exemplifies that scope 1-based emissions are a potential focal point of substantially reducing the carbon load from our direct research (see “Discussion”).

### Associated travel costs for a researcher

**Fig. 2.**
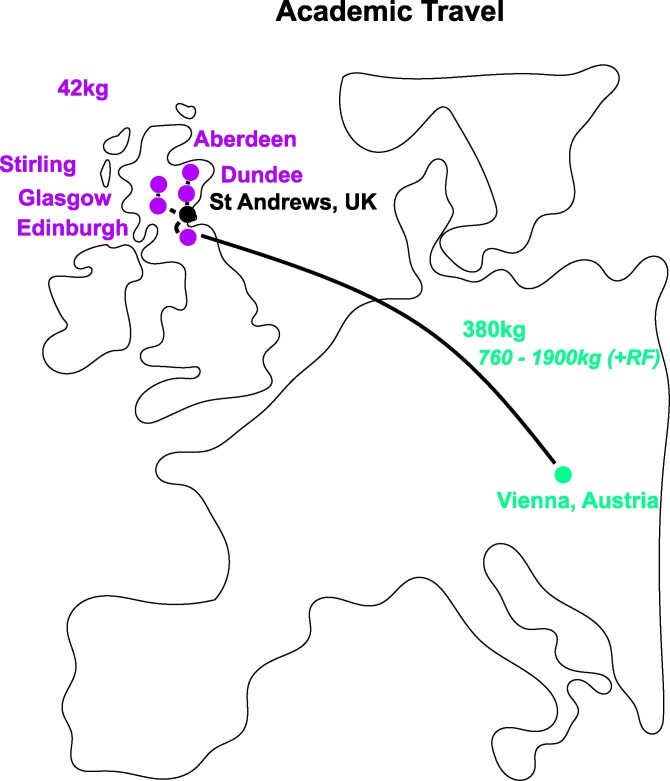
CO_2_e released via public travel for a PhD researcher in 2023 to 2024. Representation of the accrued travel costs of an individual PhD researcher throughout Scotland via national rail (Upper left, city names in Scotland) and economy air travel (lower right, Vienna, Austria) without and with (*below*) RF index factors, where relevant.

Aside from conducting research, researchers require travel for training, public engagement, and collaboration. We accounted for the travel-associated carbon emissions for 1 set of domestic and international travel for the neuroscience PhD student to provide a case study for nonresearch-related carbon costs of academia (Fig. 2). We used ICAO (ICAO 2024), a free resource for estimating the carbon intensity of travel. ICAO bases carbon emissions measurements exclusively on flight distance, cargo type, plane model, and logged fuel usage per passenger weight. However, it does not include radiative forcing (RF) factors, which encapsulate the indirect effects of greenhouse gas emissions on global warming. Succinctly, air travel induces non-CO_2_ heating effects by releasing water vapor and aerosols that amplify the heating effect by the greenhouse effect. For air travel, the RF factors are caused by contrail formation at higher altitudes during flight (Fuglestvedt et al. 2003; Lee et al. 2021).

In our case study, travel occurred both internationally via air and locally by rail. Train travel to local Scottish universities for mandatory PhD-related training and research presentation events generated 42 kg CO_2_e (Table 3). Air and associated intermediary travel to the Federation of European Neuroscience (FENS) 2024 conference in Vienna yielded 380 kg CO_2_e (Table 2). Note that the FENS conference existed beyond the June 2023 to May 2024 research life cycle analysis window but was included as exemplification of international-related travel costs for research. Using the comprehensive RF estimation range (1.0 to 4.0) made by Lee et al. (2021), we found that RF may have increased the yearly air travel carbon cost to 760 to 1,900 kg CO_2_e. Thus, the indirect cost of PhD research related to travel amounted to 442 kg CO_2_e (without RF) or 802 to 1,942 kg CO_2_e (with RF) scope 3 emissions. For comparison, the total CO_2_e emissions associated with 9 travel instances (without RF) was equivalent to 191-fold of the total experiment-related emissions (422 kg vs 2.21 kg) for the 2023 to 2024 research year. In other words, the total travel-related yearly emissions (without RF) were equivalent to the average emissions yield of ∼32 electrophysiology experiments, ∼124 dual-color calcium imaging experiments, ∼334 calcium imaging, or ∼618 behavioral optogenetics experiments conducted in South Scotland.

**Table 3.** Travel-related CO_2_e costs for PhD-related travel for training and conferences.

Route	Method	Instances (2023 to 2024)	Yearly CO_2_e (kg) without RF	Yearly CO_2_e (kg) with median RF
Leuchars–Edinburgh	Train	2	11.60	11.60
Leuchars–Stirling		1	9.68	9.68
Leuchars–Aberdeen		1	8.54	8.54
St Andrews–Leuchars	Bus	4	7.57	7.57
St Andrews–Dundee		1	4.15	4.15
Edinburgh–Frankfurt–Vienna	Plane	1	380.00	1,330.00

### Known unknowns: the international cost of *Drosophila* science

**Fig. 3.**
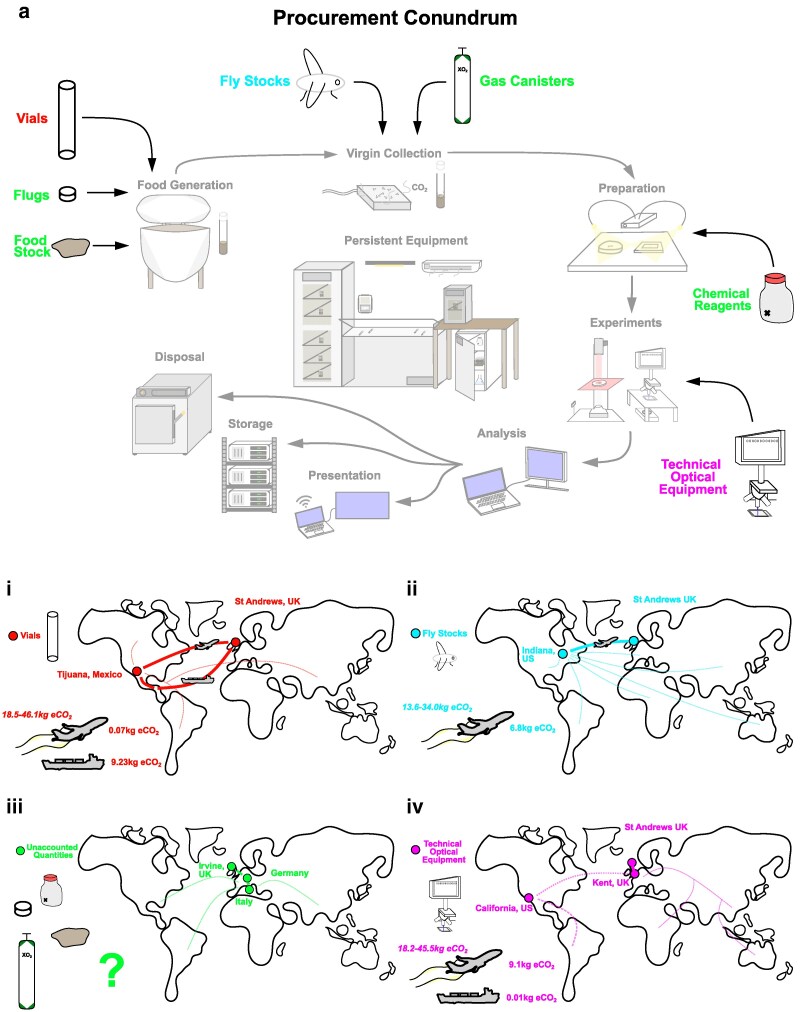
Scope 3 procurement emission estimations for *Drosophila* biological research. a) Aside from emissions produced by experimental research and persistent equipment, additional resources also have associated greenhouse emissions at the main stages of research: (i) *Drosophila* vial resources are sourced (500 per order) from Mexico through SLS; (ii) *Drosophila* stocks are mainly sourced from Bloomington, Indiana, United States, with estimates quoted per vial; (iii) unaccounted procurement resources: chemical reagents are mainly sourced from Sigma with many transport centers across Europe, gas canisters are sourced from BOC with Fife-based distributors, and flugs and food resources have unknown origin sites; (iv) optical equipment from CAIRN Research Ltd (Kent, United Kingdom) or Chroma (Irvine, California, United States) with 1 of our orders consisting of 4 filters and 1 mirror. Solid lines represent parts of the transport link with CO_2_e estimates. Dotted lines represent expected but unquantified parts of the transport procurement link upstream in the procurement chain. Radiative factor (1.0 to 4.0) air travel cost estimates are quoted behind plane images in each panel in italics.

Aside from producing direct emissions (scope 1) or indirect emissions by electricity-based experimental work and building maintenance (scope 2), equipment procurement by laboratories significantly contributes to international emissions (scope 3). Here, we focused on some ubiquitous resources for *Drosophila* research (Fig. 3a) and tried to account for their indirect emissions. However, as noted previously (Achten et al. 2013), companies often reject information requests by deeming transportation routes, times, energy mixtures in manufacturing, and cost as proprietary information. Nevertheless, some information (e.g. manufacture origin, energy mixture in manufacturing, and transportation routes) was provided and is sufficient to produce a robust estimate for the carbon cost of product distribution when combined with UK GOV Emission Conversion Data (see “Materials and methods”).

First, we attempted to estimate the cost of sourcing plastic vials for our representative PhD project. Through liaison with SLS, we discovered that their basic nonrecyclable plastic vials (widely used by *Drosophila* researchers) were sourced from Tijuana, Mexico. However, we were unable to ascertain the exact method of delivery from Mexico to the United Kingdom. Thus, we instead used best approximations for distance using Google's Distance Matrix, mode of transport assumptions, and UK GOV 2024 conversion factors for CO_2_e for mode of transport (see “Materials and methods”). If delivery uses high-carbon, faster transport (e.g. air travel with short-distance HGV transfers), we estimated an upper value of 9.23 kg CO_2_e per 500 vials for transportation from Tijuana, Mexico, to Edinburgh Airport toward St Andrews, United Kingdom. If we incorporate RF estimates (Fuglestvedt et al. 2003; Lee et al. 2021), air-based procurement of fly vials rises to 18.46 to 46.15 kg CO_2_e per 500 vials. However, if delivery uses low-carbon, slower transport (e.g. bulk HGV from Tijuana to Mexico City [Benito Juarez] and cargo shipping to London, United Kingdom), we estimate a potential 132× reduction in CO_2_e (70 g) (Fig. 3a, i; Table 4). Given a single PhD student used 624 vials (June 2023 to May 2024), vial acquisition contributed 18.46 kg CO_2_e (36.92 to 92.30 kg CO_2_e with RF) if using flight-based procurement or 0.14 kg CO_2_e if using cargo-based procurement. For the laboratory as a whole, we acquired 8,500 vials in the 2023 to 2024 research period amounting to 156.96 kg CO_2_e (313.92 to 784.80 kg CO_2_e with RF) if using flight-based procurement or 1.25 kg CO_2_e if using cargo-based procurement.

**Table 4.** Procurement-related CO_2_e costs for vial and fly stocks in 2023 to 2024 research year.

Equipment	Count	Weight per item (kg)	Total estimated travel distance (HGV + flight) (km)	CO_2_e (kg) (HGV–flight)	Total estimated travel distance (HGV + cargo) (km)	CO_2_e (kg) (HGV–shipping)
Vials (500)	17	0.16	8,464	9.23	12,181	0.07
Optic filters (4 filters and 1 mirror)	1	0.06	8,287	9.11	12,532	0.01
Fly stocks (4)	12	0.08	6,317	6.79		

In addition to vials, *Drosophila* research often relies on importing flies from Bloomington, Indiana, United States, or alternative stock centers. The time-sensitive nature of transporting live flies necessitates air freight. By using UK GOV 2024 conversion data and Google's Distance Matrix distance estimations between the origin site and the final destination, we estimated a production of 6.79 kg CO_2_e (without RF) per vial ordered to St Andrews, United Kingdom, generated by cargo flight from Indianapolis International Airport to Edinburgh Airport followed by HGV travel to St Andrews, United Kingdom (Fig. 3a, ii; Table 4). Incorporating RF, fly procurement may be 13.56 to 33.97 kg CO_2_e. Importantly, we could not estimate upstream carbon emissions from research institutions donating *Drosophila* stocks to the Indiana-based store nor the precise upkeep costs (though estimates made by this paper could be cross-applied to resources at Bloomington at a future date), so the total life cycle scope 3 cost for *Drosophila* procurement will be larger. Nevertheless, a single PhD student ordered 48 new *Drosophila* stocks from Bloomington amounting to 81.48 kg CO_2_e (162.96 to 407.40 kg CO_2_e with RF) for the June 2023 to May 2024 period.

Our research utilized optical equipment that we often continuously source from suppliers like CAIRN Research Ltd and producers like Chroma. While we were unable to acquire information about production and distribution costs from the producers of our cameras, filters, and lenses, CAIRN Research Ltd did release information about distribution centers and modes of delivery for our 2023 to 2024 items (4 filters and 1 mirror, 0.08 kg; Table 4). Our optical orders were sourced from the CAIRN Research Ltd facility in Kent, United Kingdom, by HGV to St Andrews, United Kingdom. CAIRN Research Ltd procures the optical equipment from the manufacturer intermediate Chroma in California, United States. Similar to our methodology for vial procurement estimation, we used publicly available cargo route information and UK GOV 2024 conversion factors to estimate low (HGV and cargo ship: 0.01 kg CO_2_e per order) and high (HGV and cargo flight from John Wayne to Edinburgh Airport: 9.11 kg CO_2_e per order) carbon load procurement routes (Fig. 3a, iv). Incorporating RF, optical procurement may be 15.90 to 43.22 kg CO_2_e.

In contrast to our estimations of vials, optical equipment, and fly stocks, the lack of publicly available data in chemical reagent supplication (Sigma-Aldrich, Avantor by VMR, and Thermo Fisher Scientific), gas canister supply (BOC), or yeast food made any reasonable carbon estimates from those lab dependencies unfruitful. In all, we were unable to discover the prior transportation routes, presupplies, or locations of precursor materials earlier in the supply route from any of our chemical suppliers (Fig. 3a, iii). However, we did note down our yearly usage of these materials for future estimation as information becomes more publicly available (Table 5).

**Table 5.** Yearly procurement quantity for 1 PhD student.

Equipment	Yearly used mass (g)	Quantity	Provider	Serial/batch code
NaCl	157.50	<1	Sigma-Aldrich	S9888-1KG
		<1	VMR	21A144160
KCl	7.45	<1	Sigma-Aldrich	P9333-500G
		<1	VMR	14I170013
CaCl_2_ × 2H_2_O	5.90	<1	VMR	14G090030
MgCl_2_ × 6H_2_O	16.25	<1	Sigma-Aldrich	M2670-500G
		<1	VMR	14H18004
TES buffer	22.95	<1	Sigma-Aldrich	T1375-100G
		<1	Thermo Fisher Scientific	B21819.30
Sucrose	246.50	<1	VMR	201284121
Gas canister	39,000	6	BOC	BOC UN1013 EC 204-696-9
Yeast food	6,240			
Flugs	624	<1	SLS	FLY1198

Overall, total equipment procurement for the 2023 to 2024 year for a single PhD student equated to 81.63 to 542.92 kg CO_2_e scope 3 emissions costs. In effect, the median year's procurement-related emissions (230.65 kg CO_2_e) were equivalent to 363× of the total experiment-related emissions cost. Altogether, laboratory persistent operations, experimental load, individual academic-related travel, and procurement totaled at <2,502 kg CO_2_e for the 2023 to 2024 year.

### Research costs across space and time

**Fig. 4.**
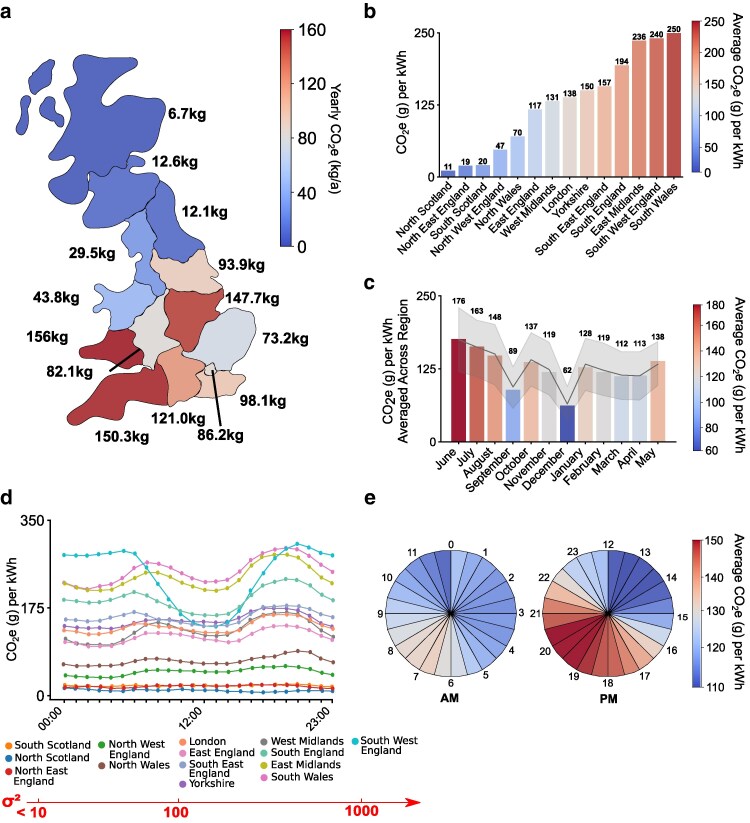
The carbon cost of research by region, month, and time of day in 2023 to 2024. a) The yearly carbon costs of 1 researcher and the total laboratory costs (total 700 vials per month) in different regions of the United Kingdom. b) The average CO_2_e per kWh generated from each region of the United Kingdom from June 2023 to May 2024. c) The monthly CO_2_e per kWh averaged across all regions from June 2023 to May 2024 as recorded by the UK National Grid. The gray line represents the average trend across all regions of the United Kingdom, while the shaded gray region shows the standard error. d) Variation in the average CO_2_e per kWh for different UK regions with time of day, with ranked variance of the regions listed below the time series plot. e) The average CO_2_e per kWh for each 30-min segment on average across regions and months in the United Kingdom from June 2023 to May 2024 as recorded by the UK National Grid.

The carbon intensity of research varies widely depending on the geographical region where it is conducted due to differing energy mixtures (e.g. fossil fuel vs renewable energy mixtures) and energy demands (e.g. household density and industry demands) across different portions of the UK electricity grid (Fig. 4a and b; see “Materials and methods”). Between June 2023 and May 2024, we estimated that persistent equipment and experimental work together produced 12.6 kg CO_2_e from mains electricity in South Scotland. Applying different regional kWh-to-CO_2_e conversion factors to the total energy consumed by persistent equipment and experimental work yields large differences in estimated CO_2_e emissions. For instance, all else being equal, conducting our research within North Scotland (e.g. Aberdeen) could have reduced total CO_2_e emissions by a factor of 1.87 (to 6.7 kg CO_2_e). In contrast, had our research consumed electricity supplied to South Wales, it would have released 12.3× more CO_2_e (156 kg CO_2_e) than we produced in South Scotland. The largest difference is between North Scotland and South Wales, with on average 23.1× more CO_2_e emissions in South Wales compared to North Scotland.

Aside from geography, the month and time that experiments are conducted also affect carbon emissions. For instance, an electrophysiology dataset (*N* = 10 preparations, 2 h per preparation) collected in South Scotland in December may produce 265 g CO_2_e, whereas generating the same dataset in June could result in 2.8× higher carbon emissions (750 g CO_2_e) (Fig. 4c).

Similarly, performing experiments at different times of day results in large variations in CO_2_e (Fig. 4d and e). For example, performing a dual-color calcium imaging experiment (1 h) at 13:00 may produce 11.3 g CO_2_e, whereas conducting the same experiment at 19:00 produces on average 36% more CO_2_e (15.5 g). However, temporal variation in CO_2_e per kWh is greater in some regions (e.g. Southwest England, *σ*^2^ = 3,505.2 g CO_2_e per kWh) than others (e.g. South Scotland, *σ*^2^ = 1.9 g CO_2_e per kWh), largely due to differing proportions of renewable resources in regional energy mixtures.

Taken together, these results highlight that timing and geographical location can substantially affect the scope 2 carbon cost of research activities. Careful consideration of the times at which energy-intensive experiments or analyses are conducted, for example, can yield measurable improvements to sustainability, especially in regions with high temporal variation in the carbon intensity of electricity.

## Discussion

Here, we exemplify how students can document their emission scope costs of their research through life cycle reporting, resolving information gaps in sector-specific sustainability and thus becoming actively engaged in sustainability. By showing the CO_2_e emissions associated with a variety of biological techniques widely used beyond the field of *Drosophila* research, we demonstrate how individual reporting can produce measured, targeted focal points for emissions reduction. For a year's worth of PhD student experimental work, travel, and procurement, we conservatively accounted for a released 542 kg CO_2_e equivalent to 3% of the median UK household's yearly CO_2_e (17.1 t CO_2_e) (Büchs and Schnepf 2013). Moving forward, using real-time documentation of research workload, students can catalog their emissions cost to catalyze and focus lab- and institutional-level improvements to sustainability in a pragmatic and tailored manner.

Using “cradle-to-grave” life cycle assessments exhibited in our paper, the medical and clinical sciences have taken great strides in carbon accounting (e.g. Robinson et al. 2018; Maida et al. 2024). However, the limited number of individual estimates, inconsistent methodology, and partial attempts at capturing complete life cycles signal the necessity of increased participation in carbon accounting and consistency in how emissions estimates are calculated (McGain and Naylor 2014; Purohit et al. 2021; Valls-Val and Bovea 2021; Rae et al. 2022; Robinson et al. 2023). The imminence of key climate targets and institutional hesitancy and backsliding necessitate efficient individual-driven collective action to mitigate carbon emissions across all sectors. We believe that accurate and publicly available emissions data will form a necessary component to inform and guide those actions. While a complete dynamic picture of the emissions landscape may be elusive, even a modest increase in emissions data and informed participants could be readily used to validate and refine large-scale carbon footprint models across disciplines, helping to inform public policy (Ni et al. 2018). We suggest that the current paucity of estimations can be partially remedied by engaging student researchers at all levels of career progression: a series of individual-led estimation efforts will, together with accurate and consistent carbon emissions estimates, help to capture complete research life cycles even if some researchers can individually account for the carbon produced only by specific life cycle elements.

Our emerging understanding of several disciplines' carbon footprints is often built upon a grassroots approach, whereby individual researchers and laboratories contribute their carbon footprint and employ ex ante and post hoc mitigation strategies to help the community, for example in astronomy (Martin et al. 2022), particle physics (Janot and Blondel 2022), medical imaging (Picano 2021), and high-performance computing (Lannelongue et al. 2021). Scientists have used these procedural insights to build accreditation programs, such as My Green Lab and LEAF that seek to inform and reward researchers' efforts to adopt sustainable research practices (Freese et al. 2024). While work has begun to account for carbon produced by medical neuroimaging (i.e. fMRI) (Souter et al. 2023, 2024), to our knowledge, our work is the first to account for cellular neuroscience imaging and *Drosophila*-related research using real-time regional emissions estimates. Further, we believe this is the first comprehensive report for any specific scientific animal model in research. We envisage similar maintenance costs for other organisms (e.g. mice husbandry); nonetheless, we actively encourage similar research assessments on other model organisms. Thus, our work, methods, and data are intended to plant the seed for further carbon accounting by other disciplines of neuroscience and science, technology, engineering, and mathematics (STEM), across institutions and geographies. Importantly, we envisage PhD-level, individual-driven, lab-focused carbon accounting building on attempts to accurately carbon footprint higher-education institutions (e.g. Valls-Val and Bovea 2021), to generate an actionable foundation for institutional and government policy for our domain of STEM.

### Methods of carbon accounting

Differences in the methods that authors use to account for carbon emissions have produced a large variability in estimates (Pandey et al. 2011). Naturally, there are sensible justifications for adopting coarse estimates when more precise estimations (e.g. equipment-specific energy consumption and regional electricity carbon intensity) are unavailable. Complementing Souter et al.’s (2023) carbon estimation approach that tracks mains electricity-related emissions using Electricity Maps (https://app.electricitymaps.com/map), we utilized the National Grid-based Carbon Intensity API (https://carbon-intensity.github.io/api-definitions/#carbon-intensity-api-v2-0-0) to demonstrate a more precise method of carbon accounting. However, at the time of writing, region-specific data were restricted to only a few areas (e.g. UK counties and some US states). As climate targets near and international pressure mounts, we predict that demand for APIs like Carbon Intensity will increase, and we hope that members of the community will advocate for local governments and private providers to publicize energy generation mixes so that developers can satisfy the need for regional precision in carbon accounting. Focusing on regional precision can enable the research community, especially future student carbon accountants, to disentangle the sources of variability in carbon footprint estimates and thus be better able to formulate effective mitigation strategies. We retrospectively accounted for our experimental CO_2_e load by registering the day and quantity of experimental types conducted based on saved data. To expedite our methodology of carbon accounting, we envisage the creation of an integrated software environment that enables logging laboratory activities, logs real-time energy use, directly communicates with country-specific grid APIs, and appropriately delivers experimental CO_2_e information. Already, we have begun to create the template of this software with WillCO_2_st. We envisage carbon appendices identifying and developing more methods and tools to increase the accuracy, ease of use, and reliability of individual reporting.

### Mitigation strategies: suggestions for improving researchers’ sustainability

For individual researchers, carbon mitigation can be readily achieved by preemptive strategies, which seek to avoid releasing anticipated emissions, while collective action through institutions that handle sequestration strategies, which aim to capture the equivalent of previous and unavoidable emissions. Assessment of a carbon footprint is often paired with an equivalent institution-led sequestration comparative (Elli et al. 2024). However, sufficient carbon sequestration is a complex demand with factors like tree species, the plantation ecosystem, climate change, biodiversity, and soil ecology all contributing to varying mitigation success (Woodward et al. 2009; Abbas et al. 2017; Vacek et al. 2023). In addition, the rise of business models around reforestation commonly incentivizes short-term sequestration followed by biomass extraction, which can render the mitigation strategy effectively redundant or unreliable. Alternative sequestration methods (e.g. carbon capture and storage) are often too expensive for individuals and university institutions to participate in the short term to reach 2030 to 2050 climate targets, especially considering the financial tradeoff made against more solvent renewable-led investment for emissions mitigation (Sgouridis et al. 2019). While postrelease mitigation is necessary to combat decades of overrelease of greenhouse gases, the complementary, more robust, and immediately accessible method of mitigation is preemptively reducing activity emissions and procurement demands, leaving sequestration efforts to capture historic and unavoidable emissions via large private and government-led initiatives.

Like with most human decisions, assessing and knowing the quantity associated with a harm or benefit can be an additive, sufficient motivating factor for action (Zhang et al. 2019) with our emissions reporting being instrumental for 4 large-scale emissions reduction in our laboratory and research. Firstly, our work revealed unexpectedly high-energy consumption by persistent climate control systems due to ageing, inefficient equipment. This has motivated us to remove or acquire more energy-efficient equipment with effects we can quantify and report consistently in the future. Secondly, we have reduced the disproportionately high emissions produced by *Drosophila* anesthetization through careful planning and exploring non-CO_2_ options. While we expect variability in anesthetization-based emissions estimates (i.e. sorting volume, type of biomarkers, and experience), considering the wide usage of *Drosophila*, the scale of CO_2_ release by the *Drosophila* community will be considerable. According to FlyBase's Fly Lab list (https://wiki.flybase.org/wiki/FlyBase:Fly_Lab_List), there were 2,074 active *Drosophila* laboratories as of December 2024. Considering we estimated *Drosophila* anesthetization and sorting released 4 kg CO_2_e per researcher, if we conservatively project an average *Drosophila* laboratory size of 3 researchers per lab, this may amount to 25,000 kg CO_2_e per year, which, according to ICAO's flight emission calculator, is equivalent to 40 return economy trips from Edinburgh to New York's JFK airport. For *Drosophila* researchers, this could take the form of anesthetization use of cold anesthetization, obvious biomarkers to reduce sorting time (e.g. curly wings), prioritizing sorting after eclosion, and using physical barriers to contain released CO_2_ close to the pad, all minimizing the need for repeated CO_2_ release. Thirdly, our own life cycle assessment has induced significant changes to our own lab research practices, including elimination of the new purchase of fly vials through using rewash-recycle protocols (Challiner et al. 2025). Fourthly, we have consciously re-evaluated how we attend conferences that necessitate large levels of air travel with early-career lab members (e.g. PhD students) now taking precedence over senior members (e.g. Principal Investigator) for conferences that necessitate large levels of air travel and prioritizing nonair travel where financially feasible. Each of these 4 changes—removing excess equipment, reducing direct CO_2_ release from anesthetizations, eliminating procurement costs by rewashing vials, and conference prioritization policy—focuses on the largest emissions costs for our *Drosophila* research. Carbon appendices will empower researchers to investigate and invest in appropriate, less carbon-intensive methods. The reality that carbon accounting students can act as such a positive force for sustainability change emboldens not just the importance of recruiting and training students to investigate and take sustainability but also, especially in research-specific domains like *Drosophila*, how scientific research can be made readily quite sustainable. Indeed, *Drosophila* research is foundational and pivotal to our understanding of biology; we must not abandon research just because it may be carbon-intensive, but we must manage this information–sustainability conflict by empowering systems to drive forward emissions reduction and general sustainability.

Efforts to account for greenhouse gas emissions across disciplines have historically used nonregional, general kWh-to-CO_2_e conversion rates that have contributed to wide variation in reported emissions. Here, we clearly demonstrate the impact of geography and time when calculating emissions estimates. Naturally, while the influence an individual researcher can have on institutional policies is conceptually limited, we foresee using carbon appendices as a tool for creating awareness and action as 1 empowering route to increase scrutiny and collective action to improve institutional sustainability. The fact that changing our lab's location within the United Kingdom could increase our annual research carbon footprint by an order of magnitude raises questions about the best geographical location for high-energy research during the transition to 100% renewable energy. Importantly, large-scale relocation of high-energy research to a currently greener region would evidently create diminishing returns if energy supply and storage were inelastic. Notwithstanding, medium-term large-scale decarbonization efforts of national grids could be complemented by short-term considerations of regional variation in energy mixtures when flexibility in the location of research or facilities exists.

While our calculations assumed electricity from the National Grid, many institutions have invested considerable effort into in-house generation of renewable, low-emission energy (Adedeji et al. 2019; Vaziri et al. 2020). This does, however, raise ethical questions concerning the link between financial privilege and the ability to invest in energy solutions: care must be taken to encourage creative, collaborative emissions mitigation strategies that are not restricted to select institutions. Indeed, even the production of low-emission energy is not immediately net-neutral; emissions are expended in construction resources, maintaining infrastructure, and transport emissions aimed to provide “greener” in-house supply (e.g. from solar power; Wu et al. 2021). Such ethical ramifications of funding allocations and research development are analogous to treatment-vs-waste questions reported in medical carbon accounting (Thiel and Richie 2022). While we cannot resolve the ethical question, we do offer effective suggestions on research-level preemptive mitigation routes applicable to all institutions regardless of geographic, economic, or social constraints.

### The procurement conundrum

We used a bottom-up approach to estimate the scope 3 footprint associated with material procurement (“cradle-to-gate”) for our research. A significant effort has been invested by the medical and adjacent community to account for material procurement costs at both the individual and institutional levels (Clabeaux et al. 2020; Montoro et al. 2023; Quann et al. 2023; Maida et al. 2024). Like other carbon accounts (McGain et al. 2020; Maida et al. 2024; Souter et al. 2024), we used government-reported statistics on fuel conversion alongside reasonable approximations of the mode and route of transport. In the case of vial and fly provision, we acquired information about product assembly, location of distribution centers, weight of shipped products, and amount of recyclable materials both via public means and by interacting with procurer sustainability representatives. However, necessary data concerning product manufacture, assembly, and distribution were often held back due to proprietary concerns. Specifically, we were unable to ascertain the following: (i) the exact mode of transport used in subparts of the distribution chain; (ii) the complete distribution route; (iii) the location, number, or energy demands of prior routes in the distribution chain (i.e. raw resource procurement); (iv) the energy mix of the manufacturer factories; or (v) the manufacturer-level mitigation strategies. While we can make robust travel cost estimations if we know the start point of the distribution chain, the precision of the account is significantly impaired by a lack of industry information and transparency (Stenzel and Waichman 2023). In some instances (e.g. gas cylinders, chemical reagents, and cotton flugs to seal *Drosophila* inside vials), we were unable to acquire any information related to procurement due to a lack of response to information requests. Nevertheless, the majority of distributors we contacted (3 of 5) were approachable, enthusiastic, receptive, and open to information requests, demonstrating how overcoming the initial energy barrier of reaching out to procurers can reveal novel and utile information. For instance, alongside learning about the procurement routes from the Bloomington *Drosophila* stock center, we also learned the total volume of *Drosophila* distribution to laboratories worldwide in 2023. The future must see both procurement institutions and researchers attempt to track, estimate, and record carbon-related procurement information so that we can better focus and evaluate emissions mitigation targets. Researchers are capable of only so much when provided with incomplete information. Institutions must report on upstream procurement (e.g. routes, methods, and loads from primary producers) as well as downstream procurement information (e.g. routes, methods, and loads to consumers). The manufacturers and distributors that are first-movers in publicly accessible and accurate emission-related data will not just gain a significant foot in the carbon net-neutral landscape but will also enable researchers to act as changemakers by making active choices to support more sustainable distributors and manufacturers, creating a race to openness by suppliers and distributors in and beyond academia.

The highly connected, dynamic, and demanding landscape for procurement often results in agents involved in the supply chain having access to precise, accurate, and highly useful information. However, the sheer volume of data and the lack of obvious public uses for supply chain metadata (e.g. number of orders, user's location, and frequency of orders) often mean that it is not made publicly accessible, increasing the energy barrier for widespread, individual-led carbon accounting. For instance, direct contact with the Bloomington *Drosophila* stock center yielded disclosure of 2023 outflow information for domestic and worldwide *Drosophila* laboratories (e.g. 171,426 vials, 17 vials per order, single orders ranging from 1 to 200 vials, and 50% of US orders to 50% international orders). This exemplifies how individual-led action can yield metadata from procurers that can help to refine and scale up estimates of carbon footprints from the researcher to the community and international levels.

Moving forward, we hope that community-driven action rising from carbon appendices and student participation will foster a culture of openness among our industry partners, kick-starting a race to openness on emissions data. The initiative shown by SLS and other companies demonstrates how researchers and suppliers can work together to inform more sustainable research practices. Indeed, the European Union (EU) have already begun legislating for carbon accounting at a higher industry level, which will continue to see EU producers and distributors release energy and CO_2_e-related information in the coming years (Dinh et al. 2023; Hummel and Jobst 2024). Supporting these efforts is a rise in initiatives derisking and aiding stakeholders (e.g. multistakeholder initiatives) to effectively meet legislative targets (Romito et al. 2024). Overall, we envisage the work to estimate procurement costs for carbon appendices to provide a powerful market incentive for data openness among resource producers and distributors.

### The future of CO_2_e research

To reach our climate targets, large-scale grid decarbonization efforts must be complemented by technological and social innovation to improve the efficiency and accessibility of renewable resources and by increasing carbon literacy, with its attendant changes in individual behavior (Sithole et al. 2016). These actions may be hampered by institutional and individual complacency, redirection of decarbonization funding to inefficient streams, and rising dominance of high-energy industries (e.g. complexification of AI and blockchain mining for cryptocurrency) (Truby et al. 2022; Cowls et al. 2023). Even without such barriers, careful planning is necessary to avoid increases to scope 3 emissions when expanding renewable energy infrastructure (e.g. mineral mining for batteries, AI-required computer chips, renewable machinery, and system-wide heat pump adoption) with consideration for equipment longevity and recycling (Jackson et al. 2024).

Within academia, climate targets will undoubtedly be challenged by increasingly energy-intensive research methods and social resistance to short-term transition costs. Fortunately, high emission sectors, including medicine (McGain and Naylor 2014) and agriculture (Jebari et al. 2024), are leading the way in carbon accounting for informed emissions mitigation, paving the way for other disciplines to interrogate their emissions landscapes, to develop strategies to meet institutional, national, and international emissions reduction targets (Zhao et al. 2024). We believe that this effort will be most effective if individuals and institutions work together to collect accurate emissions data for efficient, equitable, and community-driven transitions to sustainable research practice. Further, we actively encourage grant writers and reviewers to facilitate sustainability improvements (e.g. equipment upgrade and recycle costs, as well as carbon offsetting measures) by providing appropriate financial space and incentives for greener science.

Empowering students to create carbon appendices to their theses can accurately and efficiently engage the next generation of researchers and workers in conceptualizing and implementing sustainability initiatives. Mirroring medical and clinical researchers, we have commenced an open-source carbon data store for *Drosophila* neuroscience research. Alongside our meta-analysis reported here, we point researchers to efforts at emissions tracking at all levels of research (Loyarte-López et al. 2020; Mariette et al. 2022) that are aiding our understanding of how different research techniques, geographies, travel demands, and scientific practices affect carbon emissions. We encourage researchers at all levels to participate in building a codex of carbon accounts that will empower the scientific community to lead the way as a positive social force for climate accountability.

## Supplementary Material

iyaf268_Supplementary_Data

## Data Availability

The data are accessible from the St Andrews PURE platform: 10.17630/03a93649-62b2-43d7-bf54-1784ce505124. Supplemental material available at GENETICS online.
