# Supplementary material for: Students as carbon accountants: calculating carbon costs of a PhD in neuroscience
Source: Genetics. 2025 Dec 17;232(2):iyaf268. doi: 10.1093/genetics/iyaf268 (PMC13181407; doi:10.1093/genetics/iyaf268)
Supplement: iyaf268_Supplementary_Data [file iyaf268_supplementary_data.zip › Figure_S1_GENETICS-2025-308360.pdf]

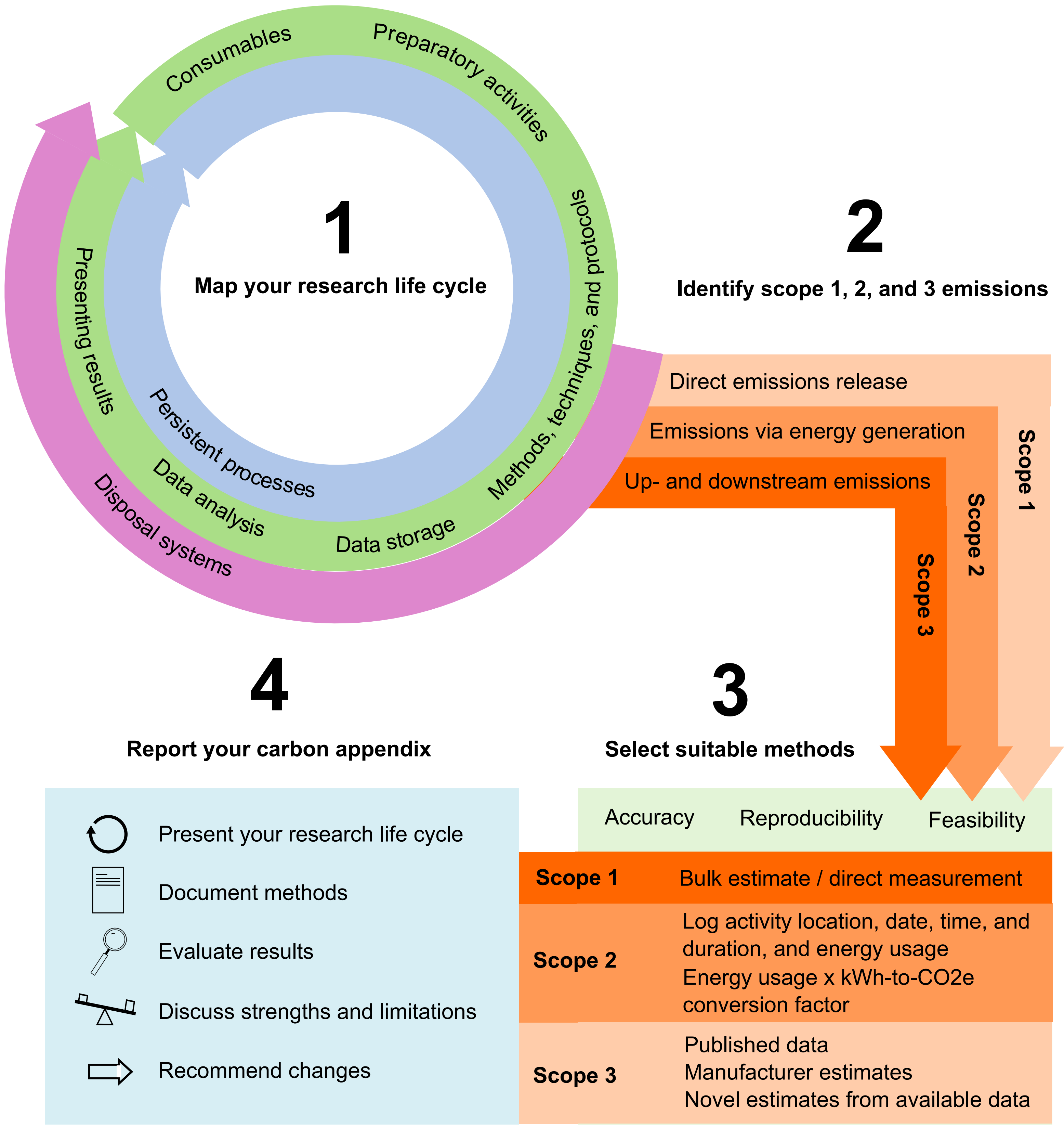
1
Map your research life cycle
Consumables
Preparatory activities
Methods, techniques, and protocols
Data storage
Data analysis
Persistent processes
Presenting results
Disposal systems
2
Identify scope 1, 2, and 3 emissions
Direct emissions release
Emissions via energy generation
Up- and downstream emissions
Scope 1
Scope 2
Scope 3
3
Select suitable methods
Accuracy
Reproducibility
Feasibility
Scope 1
Bulk estimate / direct measurement
Scope 2
Log activity location, date, time, and duration, and energy usage
Energy usage x kWh-to-CO2e conversion factor
Scope 3
Published data
Manufacturer estimates
Novel estimates from available data
4
Report your carbon appendix
Present your research life cycle
Document methods
Evaluate results
Discuss strengths and limitations
Recommend changes
