# Supplementary material for: Students as carbon accountants: calculating carbon costs of a PhD in neuroscience
Source: Genetics. 2025 Dec 17;232(2):iyaf268. doi: 10.1093/genetics/iyaf268 (PMC13181407; doi:10.1093/genetics/iyaf268)
Supplement: iyaf268_Supplementary_Data [file iyaf268_supplementary_data.zip › Supplemental_Material_and_Figure_S1_Legend_GENETICS-2025-308360.docx]

# Supplementary Information

## Guide to Creating a Carbon Appendix

Here, we provide a brief help guide with checklists to assist students with the process of creating a carbon appendix. The guidance is divided into four sections:

1. Map your research life cycle
2. Identify scope 1, 2, and 3 emissions
3. Select suitable methods
4. Report your carbon appendix
5. **Map your research life cycle**

The research life cycle refers to the sequence of activities, from procurement of consumables to waste disposal and data storage, that comprise the research process. It also encompasses the persistent processes that enable your research to take place, such as lighting and heating, as well as presentation of research output via attendance at meetings or conferences. Mapping your research life cycle can help you to identify all of the processes with which your research interacts, which together determine its sustainability.

The following questions may help you to identify the stages of your research life cycle:

- **What consumables are prerequisites for your research?** Do you require specific media, chemicals, hardware, or lab consumables such as glass slides, vials, or cover slips?
- **What preparatory activities do you need to carry out before performing experiments?** For example, do you need to conduct fieldwork, grow organisms, prepare substances, build equipment, or generate genetic lines? What sub-processes do each of these activities entail?
- **What methods, techniques, or protocols do you use?** What equipment is required for these activities?
- **What persistent processes are required by these activities?** These could include heating, lighting, or air conditioning of laboratory spaces; freezing or refrigeration of consumables; or feeding and incubation of organisms.
- **What disposal systems are in place?** What happens to the waste products of preparatory activities, experiments, and persistent processes? Are materials frozen or autoclaved? Collected for reuse, recycling, landfill, or incineration?
- **What happens to the collected data?** How are the data stored? In a central database, on a shared computer, or on hard drives? Must any physical research output, such as chemicals, organisms, or materials, be maintained after experiments? How does this occur?
- **How are the data analysed?** What hardware and/or software are used for the analysis?
- **How are results presented?** At regular meetings or conferences? What travel does this entail?

Remember that a carbon appendix does not need to be comprehensive to be useful. Documenting and reporting on even one element of the life cycle will add to emissions data and help you to identify ways to improve the sustainability of your research.

1. **Identify scope 1, 2, and 3 emissions**

After mapping your research life cycle, the next task is to identify how each element of the life cycle influences the sustainability of your research. Though the complexity of the life cycle may at first seem overwhelming, breaking down sources of emissions into scopes 1, 2, and 3 can help you to identify which quantities need to be measured in order to determine the equivalent carbon footprint of your research.

- **Scope 1:** which, if any, elements of your research life cycle are sources of direct emissions release? This could include greenhouse gases released during experiments, on-site fuel combustion for heating, energy generation, or travel.
- **Scope 2:** which elements rely on electricity generation, or other forms of purchased heating or cooling (e.g. central heating, air conditioning, microscope use, or autoclaving)?
- **Scope 3:** which elements could produce emissions up- or downstream of your research? This includes, for example, the manufacture, distribution, and disposal of consumables and equipment.

1. **Select suitable methods**

After identifying sources of scope 1, 2, and 3 emissions within your research life cycle, consider which methods are suitable for quantifying those emissions. What quantities must be known, in order to calculate emissions? Which of these quantities are known, and which are yet to be determined? What methods are conducive to obtaining reliable estimates? No two research life cycles are exactly the same; calculating the carbon footprint of your own research may well require novel methods and sector- or even laboratory-specific approaches. We suggest balancing accuracy, reproducibility, and feasibility when selecting a method: high accuracy estimates are preferable, but if such estimates require an increasingly complex and involved methodology, this may limit feasibility and reproducibility and therefore the scope for critical evaluation and comparison of results.

The following questions and suggestions may help you to identify suitable methods for quantifying emissions.

**Scope 1 measurements**

- Is there an accurate and efficient way to measure scope 1 emissions for your activities in bulk? For example, if your research involves the purchase and release of CO_2_ from canisters, can you determine your annual scope 1 emissions from the rate of canister replacement? If emissions are produced via specific reagents or combustion of fuel, can you determine the amount of emissions from the quantity of reagents or fuel used?
- If a bulk measurement is not possible, is it safe and practical to measure scope 1 emissions at their point of release (e.g. using a gas syringe, gas bladder, or other collection system)?

**Scope 2 measurements**

For scope 2 emissions arising from electricity use, you will need to determine energy usage (in kWh) and activity duration; and an estimate of the kWh-to-CO2e conversion factor for the electricity used, which depends on the geographical location, date, and time at which the activity is completed.

- How will you keep track of the geographical location, date, time, and duration of different research activities? Could you keep a detailed log of this information as you go, or will you need to determine the data post-hoc? Does your research facility use equipment booking systems that could provide this information?
- How will you measure the energy usage (in kWh) of different activities? Does your facility monitor energy usage at the building or laboratory level? Can you refer to manufacturer specifications to determine the energy usage of equipment? For which cases is it safe, practical, and preferable to directly measure energy usage power meters?
- Which kWh-to-CO_2_e conversion factor(s) are applicable for your region? You may wish to refer to the Carbon Intensity API <https://carbonintensity.org.uk/> (if in the UK) or Electricity Maps<https://app.electricitymaps.com/map/72h/hourly> (if outside the UK).

**Scope 3 measurements**

As discussed in the main text, estimation of scope 3 emissions can be challenging due to the complexity of processes involved and the difficulty of obtaining all information needed to calculate a carbon footprint. For this reason, and for comparison, it is well worth investing time to locate any previously published estimates for scope 3 emissions in your research life cycle. If estimates exist, carefully evaluate their relevance to your research. Are the emissions data case-specific, or widely applicable? Is it feasible to repeat the calculation in the context of your own research, for comparison? If so, how could you improve on the methodology?

When estimating procurement-related scope 3 emissions, consider:

- What are the procurement quantities/volumes/weights of each consumable identified in your research life cycle?
- Which companies manufacture and/or distribute each product? Do these companies provide estimates of the carbon footprints of their products? If this information is not readily available, could you contact a sustainability representative?
- If existing scope 3 estimates are not available, obtaining details of the product’s supply chain can enable a coarse estimate of its carbon cost. Consider where the manufacturer is located. Can you determine the manufacturer’s energy mixture? By what route and mode of transport is the product distributed? You may wish to use Google Maps or GPS tracking to determine the freight distance. For freight by HGV, you could then estimate emissions using publicly available km-to-CO_2_e conversion factors (UK government-reported conversion factors are available at<https://www.gov.uk/government/publications/greenhouse-gas-reporting-conversion-factors-2024>). For air freight, you could consult flight emissions calculators such as the UN’s ICAO carbon emissions calculator (<https://www.icao.int/environmental-protection/CarbonOffset>).

For travel-related scope 3 emissions, focus on identifying:

- What research-related travel do you undertake, at what frequency (e.g. weekly travel to research facilities, or annual conferences)?
- For each journey, what modes of transport were used?
- What was the distance covered using each mode of transport?

You will then be able to use the aforementioned emissions conversion factors to determine the carbon footprint of each journey. It is worth noting that many public transport providers (e.g. London North Eastern Railway and Scotrail in the UK) provide their own emissions calculators, for estimates that are specific to their fleet.

1. **Report your carbon appendix**

By this point, you will have identified and quantified the carbon footprints of elements of your research life cycle. Recall that the strength of a carbon appendix arises from the transparency of the data: by clearly communicating methods and their limitations, we can help to develop a robust carbon accounting methodology.

When reporting your findings, consider:

- Have you specified your research life cycle and which elements you tried to quantify?
- Have you clearly documented the methods you used to calculate emissions?
- Have you compared your results to any previously published estimates?
- Have you commented on the strengths and limitations of your methods, and suggested how others may improve them in the future?
- Have you included data-driven recommendations for how the sustainability of your research could be improved?
- Have you reflected on the process of creating a carbon appendix? Are there any simple changes that would make it easier to measure the carbon footprint of your research?

We provide the following question prompts, grouped under the headings from section 1, to help you start thinking about concrete ways to improve the sustainability of your research.

**What consumables are prerequisites for your research?**

- Which consumables make the greatest contribution to the carbon footprint of your research?
- Could consumables be used multiple times before reuse or disposal?
- Which consumables could be washed and reused?
- Are there suitable alternative products with a lower carbon footprint?
- Would switching to a more local manufacturer and supplier reduce the carbon cost of certain products?
- Could you reduce packaging and freight by combining orders from multiple research groups?

**What preparatory activities do you need to carry out before performing experiments?**

- What reagents, buffers, or chemical solutions do you need to prepare: can you create a mastermix, do they need to be refrigerated, can you share them amongst colleagues?
- Does your equipment need to be pre-prepared to assure quality (e.g., a microscope being on to equilibrate temperature to prevent drift?). Can you use a booking sharing system to eliminate the need for repeated pre-reparation periods?

**What methods, techniques, or protocols do you use?**

- Which of your methods, techniques, or protocols are the most carbon intensive? Why?
- Could you reduce scope 2 carbon costs by changing the time at which you carry out experiments? Is such a change feasible?
- Can you identify any unnecessary experiments that you have carried out? Could you have planned your research to avoid investing time and energy in those experiments?
- Could you adapt your protocols to allow for reuse of consumables?

**What persistent processes are required by these activities?**

- Could you turn off any equipment that is currently left on standby?
- Could fridges or freezers be maintained at higher temperatures, to reduce energy consumption?
- Are incubators, fridges, and freezers being used to capacity? If not, is there scope for multiple research groups to share these spaces?
- Is there scope to upgrade older equipment to more energy-efficient models? Is the equipment used consistently enough for the benefits of higher energy efficiency to outweigh the carbon cost of disposal of the old equipment and manufacture and distribution of the new equipment?
- Are model organisms being maintained in reasonable numbers? Is there a system in place to ensure that this is the case, e.g. a clear stock organisation system to avoid maintaining surplus copies of genetic lines?

**What disposal systems are in place?**

- What reuse or recycling systems are currently in place? Are these systems clearly communicated to users?
- Is there scope to reuse any consumables prior to disposal? For example, could glass slides, petri dishes, or vials be cleaned and reused rather than replaced?
- Is there scope to recycle any waste that is currently sent for incineration or landfill? Are there any specialist recycling services in your region that could help you to accomplish this?
- If autoclaving is required, can this be accomplished at a lower temperature, or for shorter times? Is the autoclave at full capacity for each use? Could waste be organised in a different way, to reduce the number of autoclave runs?

**What happens to the collected data?**

- Can you reduce the amount of stored data by removing redundant copies, or deleting unusable data before storage?
- Is there a case for transferring  data from storage with persistent energy costs (e.g. a central database) to hard drives or similar?
- Is there a clear plan for how long samples are retained after experiments?
- If storage of used samples incurs a carbon cost, could they be disposed of sooner?
- Is there clear guidance for when and how samples will be disposed of at the end of a research project, to prioritise storage space?
- Can any consumables be reused after sample disposal? Could samples be disposed of in a different way, to aid reuse of consumables?

**How are the data analysed?**

- Which of your methods of data analysis are the most carbon intensive?
- Are alternative softwares available that are less carbon intensive?
- Could you reduce carbon costs by changing the time of day at which analysis programs are run??

**How are results presented?**

- If you regularly present results in person or at online meetings, which mode is the least carbon intensive? Is it feasible to prioritise this mode of presentation?
- If you attend seminars, meetings, or conferences, for which events is in-person attendance particularly important? For which events would online attendance be reasonable and preferable?
- If you choose to attend a meeting in person, what is the least carbon intensive mode of transport for your journey? If this is more expensive than alternatives, is there any funding available from your institution or the meeting itself, to subsidise low-carbon travel?

### Figure S1: Steps to Create a Carbon Appendix
